# High endorsement of resource conservation in both samples alongside divergent perceived waste-sorting contexts: an exploratory comparison of two university-community study areas in Hong Kong and mainland China

**DOI:** 10.3389/fpsyg.2026.1968239

**Published:** 2026-10-09

**Authors:** Wen Ge, Shuo Li

**Affiliations:** 1 Faculty of Liberal Arts and Social Sciences, The Education University of Hong Kong, Hong Kong, Hong Kong SAR, China; 2 School of Geography and Planning, Sun Yat-Sen University, Guangzhou, China

**Keywords:** environmental attitudes, environmental knowledge, environmental psychology, Hong Kong, Chuzhou, perceived opportunities and barriers, social norms, waste sorting

## Abstract

**Introduction:**

Household waste sorting is a situated pro-environmental choice shaped not only by environmental evaluations but also by perceived norms, sorting knowledge, motivational cues, and practical opportunities or barriers.

**Methods:**

We compared item-level responses in 102 complete questionnaire records collected from two university-community study areas, one in Hong Kong SAR, China, and the other in Chuzhou, Anhui Province, China (51 records per area), using an 18-item, five-point questionnaire adapted from published studies. No archived analytic record was excluded. We report raw response distributions, two-sided Mann–Whitney tests with Holm correction across 18 items, directionally aligned Cliff’s delta with bootstrap confidence intervals, and internal-consistency diagnostics.

**Results:**

Both samples strongly endorsed the resource-conservation value of sorting, and the between-sample contrast for this item did not meet the Holm-adjusted criterion. Thirteen item-level contrasts met the Holm-adjusted criterion in the full-sample analysis. Records from the Hong Kong study area showed higher self-reported sorting-and-station knowledge, perceived regulatory constraint, publicity and education exposure, conditional willingness when bin labels were clear, and perceived facility labeling and proximity. Records from the Chuzhou study area showed stronger economic-gain motivation, whereas those from Hong Kong reported a greater household storage-space burden. Among the four original project item sets, only the environmental knowledge, publicity and education (EP) items showed high internal consistency.

**Discussion:**

High endorsement of resource conservation in both samples occurred alongside context-specific normative, informational, motivational, and perceived-opportunity profiles. These findings illustrate how context-sensitive psychological assessment can generate study-area-specific candidate intervention hypotheses for subsequent behavioral testing in larger comparative studies.

## 1 Introduction

Household waste sorting is often presented as a simple technical act, but for residents it is a daily decision embedded in social and physical environments. A person may endorse the resource-conservation value of sorting yet remain uncertain about classification rules, rarely observe others participating, or face constraints involving time, distance, and household storage. Environmental psychology therefore treats pro-environmental action as a product of person-context relations rather than environmental concern alone (Steg and Vlek, 2009; Klöckner, 2013). This distinction is especially relevant to household recycling because positive environmental evaluations may be widespread even when perceived opportunities and behavioral cues differ substantially.

The theory of planned behavior proposes that attitudes, perceived social expectations, and perceived behavioral control jointly contribute to intentions and action (Ajzen, 1991). Its overall predictive value is well supported, although the relative contribution of each component varies across behaviors and contexts (Armitage and Conner, 2001). Recycling studies have accordingly linked participation or intention to evaluations of recycling, social and moral norms, and perceived control or convenience (Tonglet et al., 2004; Chen and Tung, 2010; Park and Ha, 2014). The present questionnaire, however, did not include a complete validated theory-of-planned-behavior battery or a behavioral outcome. We therefore used the theory as a person-context framework for interpretation rather than as a causal model to be tested.

Information and the perceived socio-physical environment extend this framework in ways that are directly relevant to waste sorting. Knowledge of waste categories and collection points may reduce uncertainty, publicity may make sorting more cognitively and socially salient, and visible participation by family members or neighbors may function as a conditional cue (Xiao et al., 2017; Nigbur et al., 2010). Distance to collection points and facility availability have also been associated with recycling, whereas a perceived lack of facilities may weaken intention (Chen and Tung, 2010; Lange et al., 2014; Rousta et al., 2015). Recent studies have examined information publicity and normative pathways in e-waste recycling intentions and household waste-sorting intentions (Wang et al., 2018; Li et al., 2023). Importantly, questionnaire responses indicate how individuals perceive these conditions; they do not establish the objective quality of policies, facility networks, or services.

Research in Hong Kong has linked recycling-related responses to perceived policy effectiveness, convenience, collective-action problems, and high-rise residential conditions (Yau, 2010; Yau, 2012; Wan et al., 2014; Wan et al., 2015; Ng, 2019). Studies in mainland China have likewise reported associations of information, perceived norms, facility conditions, and policy implementation with waste-sorting responses (Xiao et al., 2017; Meng et al., 2019; Tian et al., 2022). Together, these literatures motivate a Hong Kong-mainland China comparison focused on how residents encounter normative, informational, and practical sorting conditions. Nevertheless, one study area in each setting cannot establish population-level differences: location is inseparable from the specific university-community setting and its recruitment pathway. The present comparison is therefore used as site-specific evidence for discussing broader contextual contrasts and generating hypotheses, not as a representative ranking of Hong Kong and mainland China.

Measurement presents a second challenge. Although the 18 items were adapted from published studies, borrowing item wording does not transfer the original factor structure, reliability, or cross-context measurement invariance to new samples. When internal consistency is weak, composite scores may conceal heterogeneous item content or impose a misleading common interpretation on items that do not form a coherent scale. Cronbach’s alpha alone also cannot establish unidimensionality or construct validity (Sijtsma, 2009). We therefore prioritized complete item-level distributions, effect sizes, multiplicity control, and transparent reliability diagnostics rather than constructing composite scores from the original project item sets.

Recent environmental-psychology research has tested more complex models in larger samples, including a capability-opportunity-motivation framework for waste sorting in Shanghai (Zhang, 2025), an integrated technology-acceptance and theory-of-planned-behavior model of household sorting intention (Cao et al., 2026), and an extended theory-of-planned-behavior model of source separation in southern Chile (Doussoulin et al., 2026). The archived data used here lack a validated intention or behavioral outcome, individual-level covariates, and evidence of measurement invariance, and therefore cannot support comparable path modeling. Their complementary value lies in a measurement-transparent, item-level comparison of response profiles across two university-community study areas.

Against this background, the study was organized around four theory-guided hypotheses. First, because pro-environmental values can be broadly endorsed even when situational conditions differ, we expected responses in both study-area samples to be concentrated toward agreement on the resource-conservation item (EA3), while between-sample differentiation would be more evident on items reflecting context-specific priorities and opportunities for action (H1). Second, drawing on research emphasizing legal, injunctive, personal, and descriptive normative influences on recycling, we expected the two study-area samples to differ in perceived normative guidance and responsibility related to waste sorting (H2). Third, because knowledge, publicity, and clear behavioral cues can reduce uncertainty and facilitate action, we expected the two samples to differ in perceived access to actionable sorting information and informational cues (H3). Fourth, because recycling opportunity depends on multiple external and household-level conditions, including facility access, time, storage, service experience, and economic motivation, we expected practical-context items to show differentiated rather than uniformly favorable responses across the two study areas (H4). These hypotheses were not preregistered and are evaluated within the exploratory, site-specific scope of the study; Holm-adjusted item-level tests provide the primary inferential assessment, while response-pattern diversity, the non-standard exploratory unique-pattern check, response-category diversity, ordinal dispersion checks, and internal-consistency diagnostics are reported as descriptive or measurement diagnostics.

## 2 Materials and methods

### 2.1 Study design and study areas

This cross-sectional questionnaire study was conducted in two university-community study areas in China, one in Hong Kong (114.17°E, 22.45°N) and the other in Chuzhou (118.32°E, 32.28°N), Anhui Province (Figure 1). The Hong Kong study area comprised The Education University of Hong Kong and the nearby Tianzuan residential community; the Chuzhou study area comprised Chuzhou University and the nearby Shanshui Renjia residential community. The sites were selected on pragmatic grounds. The investigator had studied at The Education University of Hong Kong and lived in the adjacent community during postgraduate training, and had previously studied at Chuzhou University as an undergraduate. This familiarity facilitated field access and contact with locally connected potential respondents. The study was designed to compare questionnaire responses collected in these two specific settings. It was not designed to estimate population parameters for Hong Kong, Chuzhou, or mainland China, although the findings are discussed in relation to the broader Hong Kong and mainland Chinese contexts.

**Figure 1:**
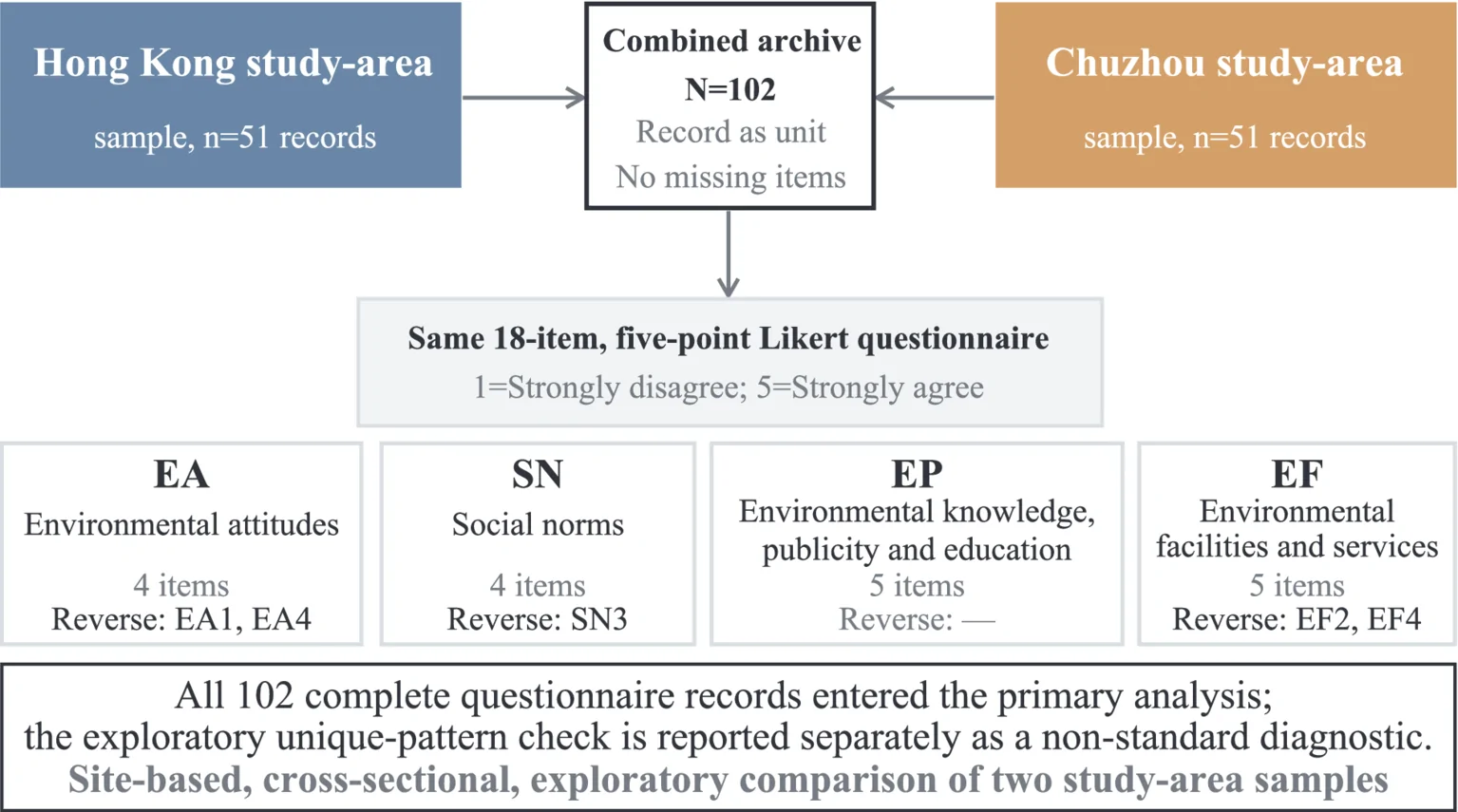
Study design and questionnaire framework. The cross-sectional comparison included 102 complete questionnaire records: 51 from Hong Kong and 51 from Chuzhou. All records entered the primary item-level analysis. The common 18-item, five-point questionnaire was organized into four project item sets: environmental attitudes (EA; four items), social norms (SN; four items), environmental knowledge, publicity and education (EP; five items), and environmental facilities and services (EF; five items). Reverse-worded items are identified in the diagram. These project groupings were retained for traceability and were not treated as validated latent scales.

### 2.2 Recruitment, eligibility, and questionnaire administration

Questionnaire data for both study areas were collected within the same fieldwork period, from 16 February 2025 to 26 February 2025. The Hong Kong and Chuzhou samples were therefore obtained within a common short survey window. At both study areas, the same investigator used a site-based intercept recruitment procedure and the same eligibility criteria, participant-information and consent procedures, and 18-item Chinese-language questionnaire. Potential respondents were approached directly at the selected university and nearby residential-community settings. Eligibility required a clear local connection to the corresponding study area, defined as currently studying or working at the selected university or residing in the nearby target community; individuals without such a connection were not included. Eligible and consenting participants completed the questionnaire through the same Wenjuanxing online survey interface using a five-point Likert response scale. The online link served as the response interface rather than as an unrestricted public or open-call recruitment mechanism. A verbatim record of the oral recruitment wording was not retained in the project archive, so identical word-for-word delivery of the recruitment approach cannot be independently verified. Nevertheless, the same investigator, eligibility criteria, participant-information and consent materials, questionnaire, response format, and administration procedure were used in both study areas. The archived analytic dataset did not contain individual-level demographic or socioeconomic variables such as age, gender, education level, income, occupation, or housing type. Recruitment-group identifiers distinguishing university students, university staff, and nearby community residents were also not retained. Consequently, demographic composition could not be compared between the two study-area samples or included as covariates in adjusted regression analyses. Because the numbers approached and declining participation were not retained in the project archive, a response rate could not be calculated.

### 2.3 Questionnaire

The instrument comprised 18 statements rated on a five-point Likert scale from 1 (strongly disagree) to 5 (strongly agree). Fifteen items—EA1-EA4, SN1-SN4, EP1-EP2, and EF1-EF5—were adapted from Meng et al. (2019). The three conditional items EP3-EP5 were adapted from Xiao et al. (2017), specifically items concerning bin labels, family-member participation, and community sorting rules. English reporting translations of the Chinese-administered items are provided in Supplementary Table S1.

The project retained the labels environmental attitudes (EA), social norms (SN), environmental knowledge, publicity and education (EP), and environmental facilities and services (EF) solely to preserve item provenance, coding, and presentation order from the project questionnaire. In the present study, these labels identify project item sets rather than psychometric dimensions, validated scales, or latent constructs. All substantive between-sample inference was therefore conducted and interpreted at the individual-item level. Because EA, SN, EP, and EF were retained only as project item-set labels rather than validated psychometric constructs, operationalization was defined at the individual-item level. EA1 operationalized the relative priority assigned to livelihood and everyday-life concerns over recycling-related environmental concerns; EA2, self-reported willingness to participate in waste sorting and recycling; EA3, endorsement of the resource-conservation benefit of sorting; and EA4, the perceived absence of a personal need to sort because little recyclable waste is produced. SN1 operationalized satisfaction associated with participation; SN2, perceived constraint from waste-sorting laws and regulations; SN3, attribution of sorting responsibility away from residents and toward government and businesses; and SN4, the conditional influence of participation by neighbors or friends. EP1 operationalized self-perceived knowledge of sorting methods and nearby recycling-station locations; EP2, perceived exposure to sorting-related publicity and education; EP3, conditional willingness to sort when bin labels are clear; EP4, the perceived facilitative influence of family-member participation; and EP5, the perceived facilitative influence of community sorting rules. EF1 operationalized the salience of economic return from selling recyclable materials; EF2, perceived time burden; EF3, perceived availability, labeling clarity, and proximity of community sorting facilities; EF4, perceived household storage-space burden; and EF5, perceived recycling-station proximity together with satisfaction with recycling services. These operational interpretations refer only to the content directly represented by each self-report item and should not be interpreted as objective measures of policy quality, facility conditions, knowledge accuracy, or actual sorting behavior. EA1, EA4, SN3, EF2, and EF4 were reverse-worded. Raw distributions retain the original 1–5 responses; for directionally aligned means, effect sizes, and internal-consistency diagnostics, these five items were reverse-scored as 6 minus the raw response.

The project retained EF4, although Meng et al. (2019) removed it from their scale during a reliability-based item deletion analysis. EP3-EP5 were assigned to the project’s EP item set after adaptation from Xiao et al. (2017), but neither source study validated the present five-item EP combination. These differences in item origin and project item-set assignment further supported item-level rather than composite-scale or latent-construct analysis.

### 2.4 Data completeness and response-pattern audit

All 102 questionnaires contained responses to all 18 items, yielding 1,836 item responses with no missing values. No record was excluded from the primary analysis on the basis of completion time, invariant responding, or similarity between complete response patterns. Questionnaire records were treated as independent observations in the item-level comparisons. The analytic archive did not contain household relationships, recruitment-group identifiers, verified personal identifiers, or other information with which to evaluate dependence between records. Residual non-independence therefore could not be assessed, and the assumption that each eligible respondent contributed only one record could not be independently verified from the archive.

Complete 18-item response vectors were also tabulated separately within each study-area sample to describe response-pattern diversity. The Hong Kong sample contained 50 distinct response vectors among 51 records (98.0%), whereas the Chuzhou sample contained 41 distinct vectors among 51 records (80.4%). Identical response vectors were not interpreted as evidence of duplicate or invalid participation because different respondents can legitimately provide the same complete set of responses. Frequency tabulation showed that, in the Hong Kong sample, 49 distinct response vectors occurred once and one occurred twice. In the Chuzhou sample, 37 distinct response vectors occurred once, two occurred twice, one occurred four times, and one occurred six times. As a non-standard exploratory diagnostic, we additionally repeated the item-level comparisons after retaining only the first occurrence of each distinct response vector within each sample. This procedure was not intended to identify duplicate records or to resolve the independence assumption. It was used only to describe how item-level estimates changed when distinct complete response vectors were given equal representation rather than contributing according to their observed frequencies. Detailed results from this diagnostic are reported only in the Supplementary Figure S1.

### 2.5 Statistical analysis and data visualization

All analyses were performed in R 4.2.3. Raw Likert-category proportions were reported before any reverse scoring. For each item, the samples were compared using a two-sided Mann–Whitney/Wilcoxon rank-sum test with continuity correction. The 18 items formed one multiplicity family, and the primary inferential criterion used Holm adjustment (Holm, 1979). Raw *p* values and Benjamini-Hochberg-adjusted *p* values were retained in the source data, but inferential statements in the manuscript were based only on the Holm-adjusted results.

Between-sample differences were quantified using Cliff’s delta (δ) (Cliff, 1993), calculated from the directionally aligned item scores. Reverse scoring of the five reverse-worded items served only to make higher scores consistently indicate less negation, rejection, or burden; it did not imply that every higher response was normatively better. A positive δ indicated a higher aligned response in the Hong Kong sample, and a negative δ indicated a higher response in the Chuzhou sample. Percentile 95% confidence intervals were obtained from 10,000 within-sample bootstrap resamples with replacement. These intervals were unadjusted descriptive intervals; inferential significance labels were determined by the Holm-adjusted tests across all 18 items.

As a secondary exploratory description of response-category occupancy, we calculated the standardized Gini-Simpson response-category diversity index, H = (1 – Σp^2^)/0.8, where p is the proportion of responses in each of the five raw Likert categories. Higher H indicates that responses are distributed across a larger or more even set of response categories. Because H treats the response categories without regard to their ordinal distance, it is not an ordinal dispersion statistic and cannot distinguish, for example, concentration in adjacent categories from concentration at opposite ends of the response scale. It was therefore used only to describe category occupancy and concentration and was not interpreted as psychological polarization, response extremity, or fragmentation of social values. To provide ordinally sensitive descriptive checks, we also calculated the sample standard deviation and interquartile range for each item in each study-area sample. Standard deviation reflects numerical distance under the conventional 1–5 coding of the Likert categories, whereas the interquartile range reflects the ordered spread of the central 50% of responses. These measures were used only to examine directional agreement with the exploratory category-diversity results and did not generate an additional family of significance tests.

For the four original project item sets (EA, SN, EP, and EF), raw Cronbach’s alpha was estimated after reverse scoring in the pooled data and in each sample (Cronbach, 1951), solely as an internal-consistency diagnostic to assess whether grouping-level interpretation was defensible. Record-level 95% confidence intervals were obtained from 10,000 bootstrap resamples; non-estimable resamples were recorded rather than silently discarded. Item diagnostics comprised corrected item-total Spearman correlations and raw alpha after deleting each item. Because adequate reliability, unidimensionality, and cross-sample structural equivalence were not established, no composite, summed, averaged, factor, or latent scores were calculated for any of the four item sets. All inferential comparisons were performed at the individual-item level, and no regression or structural-equation model was fitted.

## 3 Results

### 3.1 Both samples strongly endorsed resource conservation but showed different raw response profiles

Raw response distributions showed strong endorsement of the resource-conservation value of waste sorting in both samples (Figure 2). For EA3, 100.0% of Hong Kong records and 90.2% of Chuzhou records selected agree or strongly agree. Directionally aligned means were 4.71 and 4.41, respectively, and the between-sample contrast did not meet the Holm-adjusted criterion (Cliff’s δ = 0.146, 95% CI [−0.042, 0.329], pHolm = 0.258). This non-significant result is not interpreted as evidence of equivalence between the two samples, and the confidence interval does not exclude all potentially meaningful between-sample differences. We therefore describe the pattern descriptively as high endorsement of resource conservation in both samples.

**Figure 2:**
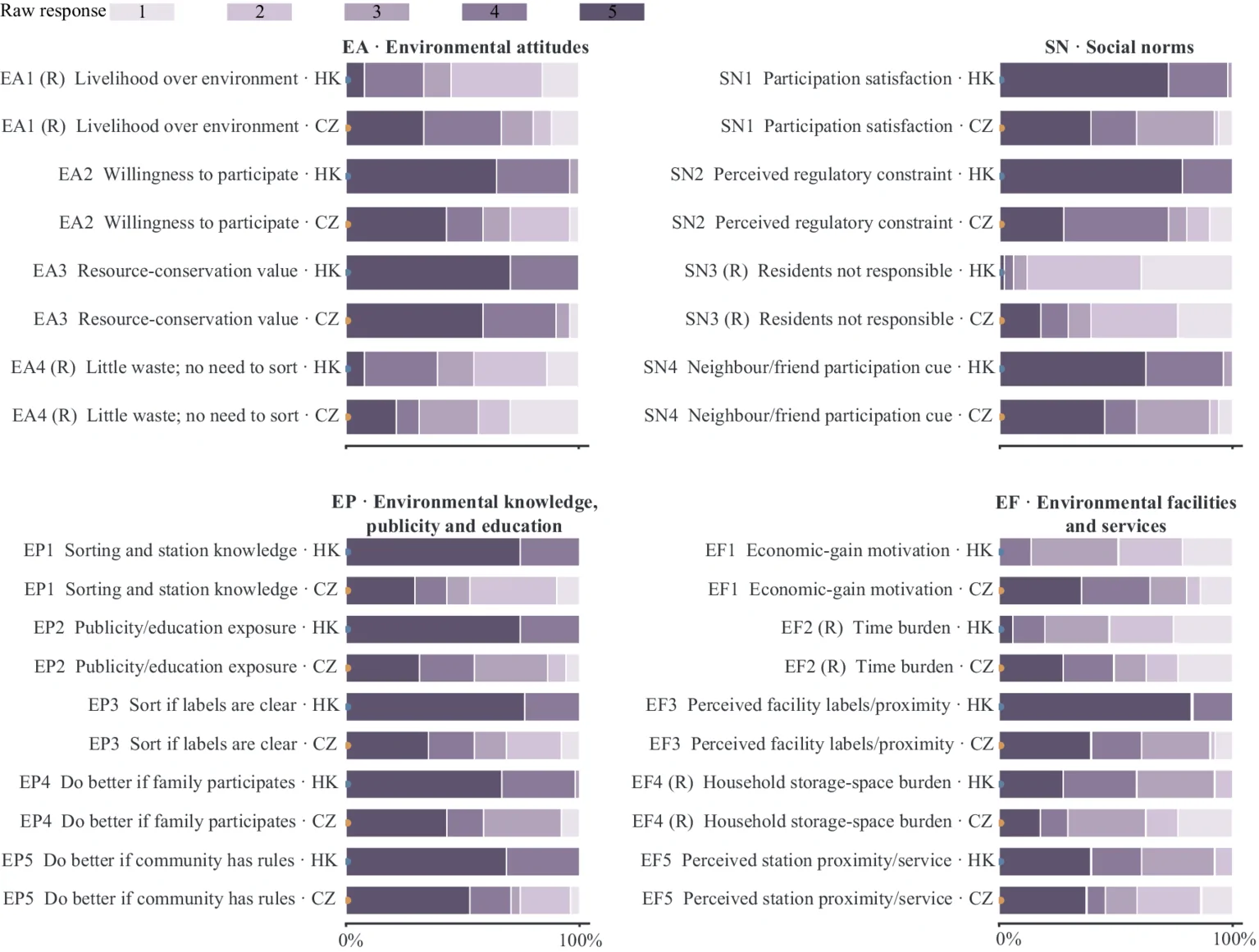
Stacked bar chart showing raw five-point Likert response distributions for 18 questionnaire items in the Hong Kong and Chuzhou study-area samples, with 51 records per sample. Items are grouped into environmental attitudes (EA), social norms (SN), environmental knowledge, publicity and education (EP), and environmental facilities and services (EF). Each 100% stacked bar displays the proportions of responses from 1 (strongly disagree) to 5 (strongly agree). Reverse-worded items are marked “R” but are not reverse-scored in this figure. The figure is descriptive and contains no inferential significance coding.

### 3.2 Item-level differences between samples

Of the 18 comparisons, 13 met the Holm-adjusted significance criterion (Figure 3; Supplementary Table S2). The largest positive contrasts concerned self-reported knowledge of sorting methods and nearby station locations (EP1; Hong Kong M = 4.75, Chuzhou M = 3.16; δ = 0.596, 95% CI [0.428, 0.750], pHolm = 3.11 × 10^−7^), perceived regulatory constraint (SN2; M = 4.78 vs. 3.71; δ = 0.569, 95% CI [0.403, 0.719], pHolm = 7.05 × 10^−7^), exposure to publicity and education (EP2; M = 4.75 vs. 3.67; δ = 0.546, 95% CI [0.378, 0.705], pHolm = 3.08 × 10^−6^), conditional willingness when bin labels were clear (EP3; M = 4.76 vs. 3.51; δ = 0.518, 95% CI [0.340, 0.675], pHolm = 8.88 × 10^−6^), and perceived facility labeling and proximity (EF3; M = 4.82 vs. 3.82; δ = 0.501, 95% CI [0.333, 0.659], pHolm = 8.97 × 10^−6^).

**Figure 3:**
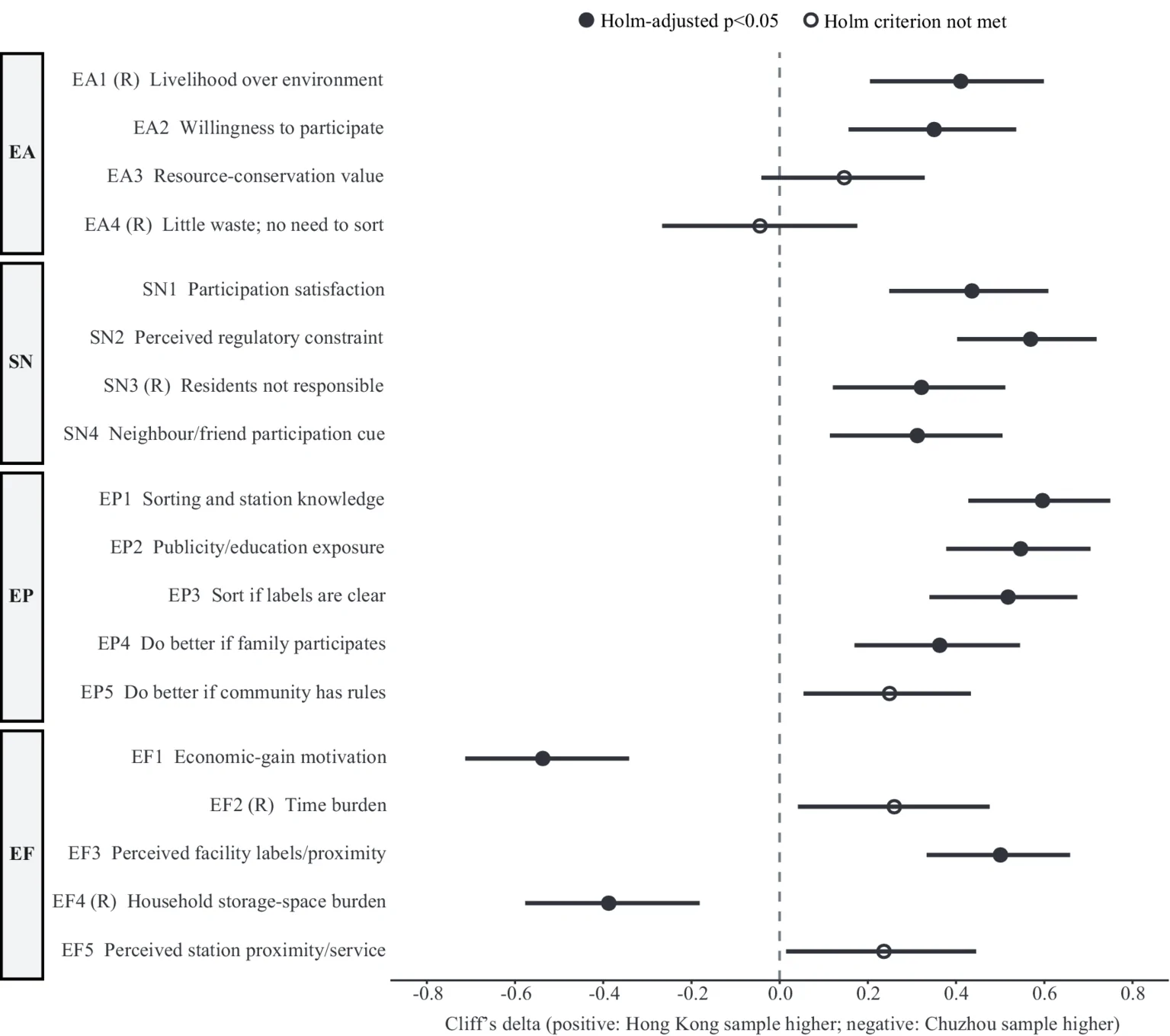
Horizontal effect-size plot showing Cliff’s delta for 18 questionnaire items comparing the Hong Kong and Chuzhou study-area samples. Items are grouped as EA, SN, EP, and EF, representing environmental attitudes, social norms, environmental knowledge, publicity and education, and environmental facilities and services, respectively. Points show effect-size estimates, and horizontal lines show unadjusted percentile-based 95% confidence intervals from 10,000 within-sample bootstrap resamples. Filled points indicate Holm-adjusted *p* < 0.05, whereas open points did not meet this criterion. Positive values indicate higher directionally aligned responses in Hong Kong, and negative values indicate higher responses in Chuzhou.

Records from the Hong Kong sample also had higher aligned scores for the reverse-scored item prioritizing livelihood over environmental issues such as waste recycling (EA1; M = 3.29 vs. 2.31; δ = 0.411, 95% CI [0.205, 0.599], pHolm = 0.00270), satisfaction with participating in sorting and recycling (SN1; M = 4.71 vs. 3.84; δ = 0.436, 95% CI [0.248, 0.609], pHolm = 2.98 × 10^−4^), and the reverse-scored statement that sorting and recycling were unrelated to residents (SN3; M = 4.20 vs. 3.37; δ = 0.321, 95% CI [0.121, 0.512], pHolm = 0.0196). These contrasts describe self-reported evaluations and perceived expectations rather than directly measured motivation or enacted responsibility.

Two significant negative effects indicated different motivational and burden profiles. Records from Chuzhou showed stronger economic-gain motivation (EF1; Chuzhou M = 3.67, Hong Kong M = 2.43; δ = −0.537, 95% CI [−0.713, −0.341], pHolm = 2.30 × 10^−5^). Conversely, after reverse scoring and directional alignment of the storage-space item, Hong Kong had a lower score (EF4; M = 2.22 vs. 3.14; δ = −0.388, 95% CI [−0.577, −0.181], pHolm = 0.00477), indicating a more prominent reported burden of storing recyclables at home.

Three additional contrasts met the Holm-adjusted criterion in the full-sample analysis: willingness to participate in sorting and recycling (EA2; δ = 0.350, pHolm = 0.00653), the cue provided by neighbors or friends participating (SN4; δ = 0.312, pHolm = 0.0196), and the conditional response to family-member participation (EP4; δ = 0.363, pHolm = 0.00477). Although the unadjusted bootstrap intervals for EP5, EF2, and EF5 excluded zero, their Holm-adjusted tests did not meet the significance criterion and they were not interpreted as primary findings. Figure 4 integrates common response levels and between-sample effects descriptively without introducing new statistical tests.

**Figure 4:**
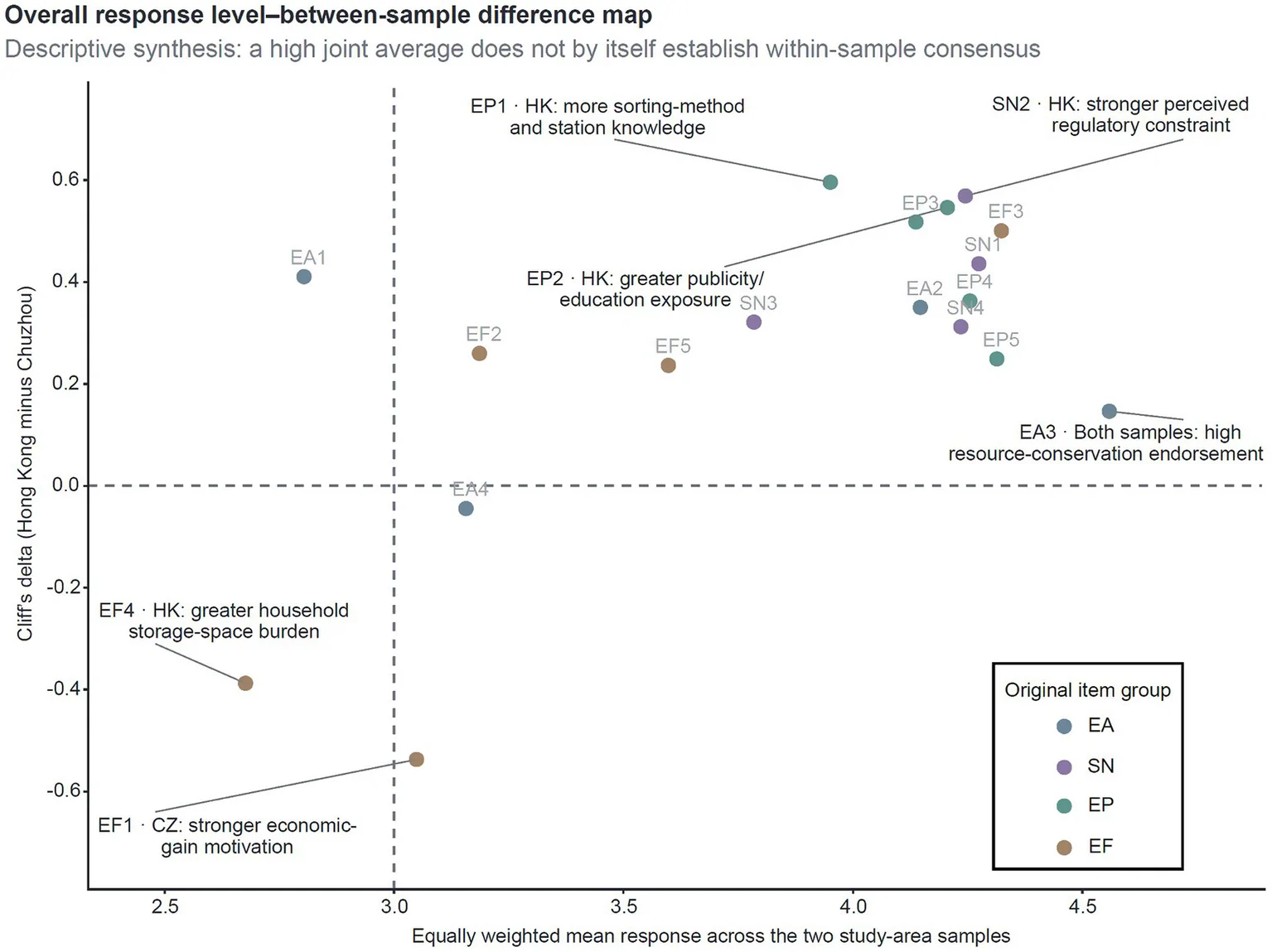
Scatterplot showing the overall response level–between-sample difference map. Each point represents one questionnaire item and is color-coded by its original item group (EA, SN, EP, or EF). The x-axis shows the equally weighted average response across the Hong Kong and Chuzhou samples, and the y-axis shows Cliff’s delta, with positive values indicating higher directionally aligned responses in Hong Kong and negative values indicating higher responses in Chuzhou. Dashed reference lines mark a mean response of 3 and a Cliff’s delta of 0. Annotations highlight selected item-level patterns. Labels such as EA1, SN2, EP1, and EF1 denote individual questionnaire item codes and are not figure part labels. The x-axis is descriptive and does not represent a validated composite score or within-sample consensus.

### 3.3 Response-pattern diversity

The proportion of distinct complete response vectors was 98.0% in the Hong Kong sample (50/51) and 80.4% in the Chuzhou sample (41/51). In Hong Kong, 49 distinct response vectors occurred once and one occurred twice, whereas in Chuzhou, 37 distinct vectors occurred once, two occurred twice each, one occurred four times, and one occurred six times. This descriptive difference does not establish duplicate participation, invalid responding, record dependence, or any specific psychological process. As a non-standard exploratory diagnostic, we additionally examined how the item-level estimates changed after retaining one occurrence of each distinct complete response vector within each study-area sample. Detailed results are provided in Supplementary Figure S1. This diagnostic should not be interpreted as validating or invalidating the primary full-sample findings, whose inferential status is determined by the analysis of all 102 archived records.

### 3.4 Exploratory response-category diversity, ordinal dispersion checks, and internal-consistency diagnostics

As a secondary exploratory diagnostic, the standardized Gini–Simpson index was used to examine differences in response-category occupancy between the two study-area samples. The associated permutation tests and Holm adjustment were used only to summarize these exploratory distributional comparisons and do not constitute an additional confirmatory family of psychological hypotheses. The primary inferential assessment of the study remains the Holm-adjusted item-level Mann–Whitney/Wilcoxon comparisons reported in Section 3.2. Detailed Gini–Simpson estimates and exploratory permutation results are provided in Supplementary Figure S2. Ordinally sensitive descriptive checks showed the same overall direction: standard deviations were larger in Chuzhou for all 18 items, while interquartile ranges were larger in Chuzhou for 15 items and equal between samples for EA1, EA3, and EF4. These results indicate broader or less concentrated response distributions in the Chuzhou sample but do not establish psychological polarization or any specific underlying response process.

Internal consistency differed markedly across the four original project item sets (Figure 5; Supplementary Table S3). In the pooled data, Cronbach’s alpha was 0.483 (95% CI [0.279, 0.628]) for EA, 0.642 (95% CI [0.449, 0.774]) for SN, 0.919 (95% CI [0.864, 0.952]) for EP, and −0.168 (95% CI [−0.566, 0.093]) for EF. EA alpha was 0.603 in Hong Kong and 0.322 in Chuzhou; SN alpha was 0.653 and 0.404; EP alpha was 0.860 and 0.895; and EF alpha was −0.204 and 0.092, respectively. Removing SN3 increased pooled SN alpha to 0.800. EF1 had a corrected item-total Spearman correlation of −0.423, and removing it increased pooled EF alpha to 0.407. Overall, the EF items clearly did not support interpretation as a common composite scale, while the low and sample-dependent consistency of EA and SN provided insufficient support for treating either item set as a unidimensional scale in the present samples. Although EP showed high internal consistency, alpha alone does not establish unidimensionality or cross-context measurement invariance. Accordingly, none of the four project item sets was used to construct a composite score, and all subsequent substantive interpretation remained at the individual-item level.

**Figure 5:**
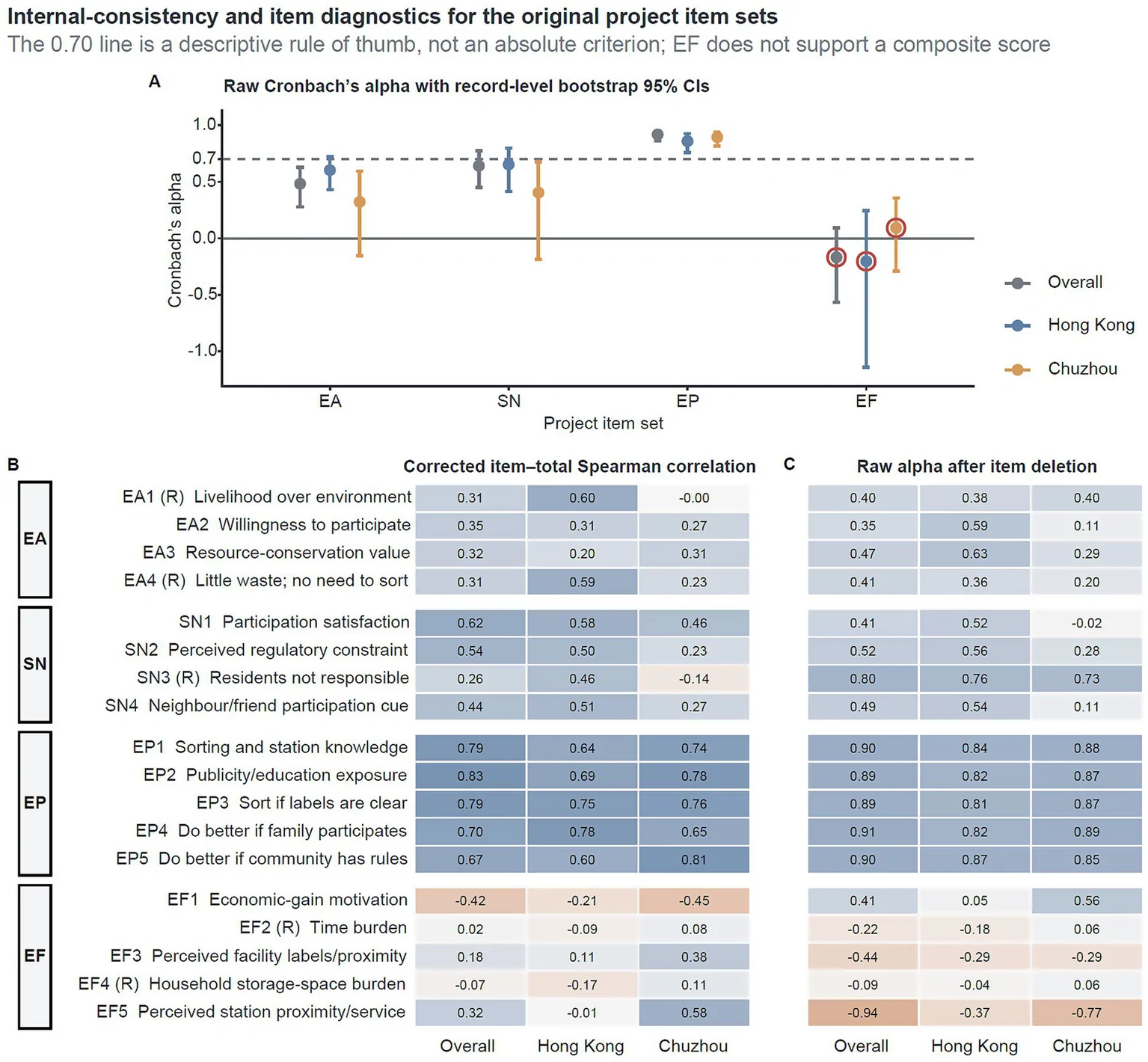
Three-panel figure presenting internal-consistency and item diagnostics for the original EA, SN, EP, and EF project item sets. **(A)** Raw Cronbach’s alpha with record-level bootstrap 95% confidence intervals. The dashed line marks α = 0.70 as a reference value. Red circles highlight the EF estimates, which showed poor internal consistency across the overall, Hong Kong, and Chuzhou samples; they do not indicate statistical significance or an additional statistical test. **(B)** Corrected item-total Spearman correlations. **(C)** Raw Cronbach’s alpha recalculated after deleting each item in turn.

## 4 Discussion

The study identified a bounded but psychologically informative contrast: both study-area samples strongly endorsed the resource-conservation value of waste sorting, while their normative, informational, motivational, and perceived-opportunity profiles differed. Thirteen item-level contrasts met the Holm-adjusted criterion in the full-sample analysis. The item-level patterns collectively illustrate how high endorsement of resource conservation in both samples can occur alongside different perceived routes to action across the two study-area samples.

### 4.1 High endorsement of resource conservation in both samples accompanies different environmental priorities

The environmental-attitude items are most informative when read as evidence about the relationship between an abstract environmental value and the priority assigned to that value when it competes with other concerns, rather than as a simple high-versus-low attitude comparison. The wider literature distinguishes general environmental concern from behavior-specific attitudes, intentions, and enabling conditions. Integrated and meta-analytic accounts indicate that values and attitudes contribute to pro-environmental action, but their influence is transmitted or constrained by perceived control, moral norms, habits, and situational costs (Steg and Vlek, 2009; Klöckner, 2013; Stern, 2000; Bamberg and Möser, 2007; Barr, 2007). In a natural experiment on curbside recycling, the provision of collection bins changed recycling and the conditions under which attitudinal variables predicted it, showing that similar attitudes can have different behavioral implications under different external conditions (Guagnano et al., 1995). This distinction is directly relevant here: EA3 captures endorsement of resource conservation, whereas EA1 and EA2 probe whether environmental concerns retain priority alongside livelihood demands and whether endorsement is accompanied by willingness to participate. The central comparison is therefore not whether one sample cared about the environment and the other did not, but how high endorsement of the resource-conservation item in both samples was accompanied by differences in competing priorities and perceived possibilities for action.

In the Hong Kong sample, all records endorsed the resource-conservation statement in EA3, indicating a strong but ceiling-concentrated value baseline. The EA1 contrast adds information that EA3 alone cannot provide: Hong Kong records were less likely to place livelihood and everyday-life concerns ahead of recycling-related environmental issues. In the present data, the coexistence of uniformly high EA3 responses and the EA1 difference suggests that environmental concern was not merely endorsed in the abstract but occupied a comparatively salient position when the questionnaire presented a priority trade-off. At the same time, EA4 did not meet the Holm-adjusted criterion, indicating that the between-sample pattern did not extend uniformly across all environmental-attitude items. EA2, by contrast, showed a higher willingness response in the Hong Kong sample in the full-sample Holm-adjusted analysis. The literature therefore helps refine, rather than inflate, the interpretation: attitudes may orient action, but their behavioral expression still depends on costs, opportunities, and behavior-specific intentions (Stern, 2000; Guagnano et al., 1995; Bamberg and Möser, 2007; Barr, 2007). The Hong Kong findings do not establish greater actual sacrifice, sorting accuracy, or participation; instead, they identify a sample in which high resource-conservation endorsement was accompanied by a more consistently expressed environmental priority. Thus, the most defensible Hong Kong conclusion is a comparatively salient value hierarchy, not a general claim that Hong Kong residents possess superior environmental attitudes.

The Chuzhou sample also showed high endorsement of the resource-conservation value of sorting: 90.2% of records endorsed EA3, while all Hong Kong records selected agree or strongly agree. The non-significant EA3 result is substantively informative but should not be interpreted as evidence of equivalence. Both samples showed very high endorsement of the resource-conservation value of sorting. However, the Holm-adjusted test and the confidence interval for Cliff’s delta do not establish equivalent response levels and do not exclude a potentially meaningful between-sample difference. Because no equivalence margin or smallest effect size of interest was prespecified, the present study does not make an equivalence claim. The strong concentration of responses near the upper end of the scale may also have limited the item’s ability to discriminate between samples. Accordingly, the most defensible interpretation is that resource conservation received high endorsement in both samples, while the present data do not establish that the magnitude of endorsement was equivalent across the two study areas. EA4 likewise showed little evidence of between-sample differentiation. Because this item combines perceived recyclable-waste quantity with a judgment about whether sorting is necessary, its interpretation may also depend on how respondents linked these two ideas. The important difference lies in the organization of that value rather than its absence. The lower directionally aligned EA1 response indicates greater endorsement of giving livelihood and everyday-life concerns priority over recycling-related environmental issues, while the larger dispersion for EA2 indicates that readiness to participate was less uniform. This pattern is compatible with models in which pro-environmental values coexist with variable situational costs and perceived control (Steg and Vlek, 2009; Stern, 2000; Guagnano et al., 1995; Bamberg and Möser, 2007; Barr, 2007). In the present study, however, income, occupation, household responsibilities, and access to opportunities were not measured, so the greater heterogeneity cannot be assigned to economic pressure or any other single explanation. It could also reflect differences in item interpretation or in the relevance of recycling to university- and community-linked daily routines. Given the high endorsement of the resource-conservation item in both samples, a candidate hypothesis for future research is that reducing perceived effort, clarifying feasible actions, or linking conservation to concrete situational cues may be more informative to test than value-affirming messages alone. The present questionnaire does not establish that any of these approaches would change actual sorting behavior.

### 4.2 Regulatory expectations and resident responsibility distinguish the normative contexts

The social-norm findings are best understood as a configuration of several normative processes rather than a single normative climate. Regulatory expectations resemble injunctive or legal norms about what people should do; observations of neighbors and friends provide descriptive cues about what people appear to do; rejection of the claim that sorting is unrelated to residents reflects personal responsibility; and satisfaction with participation may provide an affective reinforcement for norm-consistent action. Field research on kerbside recycling found that personal norms, descriptive norms, self-identity, and neighborhood identification made distinct contributions to intention and observed behavior (Nigbur et al., 2010). Research integrating norms with convenience likewise found that social, legal, and descriptive norms can help activate personal recycling norms, while convenient collection remains independently important (Berglund et al., 2022). These studies clarify why the present SN items should not be collapsed into a single explanation. The key issue is whether regulatory expectation, personal responsibility, peer visibility, and satisfaction form a mutually supportive perceived context and how these components are expressed across the two study-area samples.

For the Hong Kong study area, the large SN2 difference indicates that regulatory constraint was more psychologically salient, while the SN3 difference indicates a stronger rejection of the idea that sorting is unrelated to residents. The simultaneous SN1 difference adds a motivational-affective element: participation was associated with a greater sense of satisfaction. In the present data, these three items form a coherent pattern in which external expectation, personal responsibility, and positive evaluation of participation were jointly prominent. This pattern is compatible with Hong Kong studies linking perceived policy effectiveness, collective action, willingness to support recycling measures, and multi-family dwelling arrangements to recycling responses (Yau, 2010; Yau, 2012; Wan et al., 2014; Wan et al., 2015; Ng, 2019). SN4 also met the Holm-adjusted criterion in the full-sample analysis, indicating a between-sample difference in the neighbor-or-friend participation cue. Because SN4 captures descriptive peer influence whereas SN2 and SN3 concern regulatory expectation and personal responsibility, these findings should be interpreted as distinct normative processes rather than as evidence of a single uniformly stronger form of social influence.

In the Chuzhou sample, lower SN1-SN3 averages, together with greater response-category diversity and broader ordinal spread in the descriptive checks, indicate that normative responses were less concentrated across the sample. Prior mainland Chinese studies have related policy implementation, information publicity, moral norms, and perceived normative pathways to sorting responses and intentions (Li et al., 2023; Tian et al., 2022; Zhang, 2025; Cao et al., 2026). Recent evidence from 720 valid questionnaires collected in Beijing showed that stricter waste-sorting supervision was associated with a higher probability of reported sorting participation, with awareness of sorting significance, standards, and policies identified as potential pathways (Li and Zhou, 2026). Their study also identified heterogeneity according to age and income. These findings provide a useful contemporary context for the present SN2 result, in which regulatory constraint was more salient in the Hong Kong study-area sample, and for the accompanying differences in sorting knowledge and publicity exposure. However, the two studies differ substantially in sampling frame, questionnaire design, and outcome measures, and the present study did not collect the demographic covariates required to evaluate comparable age- or income-related heterogeneity. The comparison should therefore be interpreted as contextual convergence in the relevance of regulatory and informational processes rather than as evidence that the present samples are representative of broader urban populations. Placed against that evidence, the present pattern may reflect uneven exposure to rules, differences in which reference group is psychologically relevant, or variability in whether sorting is framed as an individual, university, household, or community responsibility. These explanations remain hypotheses because the questionnaire did not identify respondents’ reference groups or measure policy contact and normative feedback. These item-level differences should not be interpreted as proof that rules were absent or that local residents lacked responsibility, because the questionnaire assessed perceived normative conditions rather than objective regulatory implementation or enacted civic responsibility. A more precise next step would be to measure source-specific normative exposure and experimentally compare rule clarification, feedback on collective participation, and carefully designed descriptive-norm messages. Thus, the Chuzhou findings support a conclusion of less consistently perceived normative guidance, not a citywide deficit in civic responsibility or regulation.

### 4.3 Knowledge, publicity, and clear labels form the strongest contextual contrast

The EP findings suggest that information matters most when it becomes actionable at the point of sorting, rather than when publicity is treated as exposure alone. Existing intervention evidence supports this distinction. A systematic review and meta-analysis of validated field interventions found that social modeling and environmental alterations were among the most effective approaches, indicating that information often works within a broader behavioral setting rather than as an isolated message (Varotto and Spagnolli, 2017). In a household field study, written leaflets did not significantly improve food-waste separation, whereas installing in-home sorting equipment produced a substantial and sustained improvement (Bernstad, 2014). Reviews of urban household separation similarly emphasize specific communication, public education, and adaptation to local systems and social contexts (Knickmeyer, 2020). These findings provide a useful interpretation of the present EP items: perceived knowledge of methods and stations, exposure to publicity, and clear labels can operate as an informational system that reduces ambiguity, but they are not equivalent measures and cannot by themselves establish learning or behavioral change.

The Hong Kong sample showed the clearest information-related profile. EP1, EP2, and EP3 were among the largest item-level effects in the full-sample analysis and met the Holm-adjusted criterion: records indicated greater self-reported knowledge of sorting methods and station locations, more exposure to publicity and education, and greater conditional willingness when bin labels were clear. In the present study, the convergence of these three findings is more informative than any single item. It suggests a perceived information environment in which knowing what to do, encountering repeated communication, and recognizing an actionable cue were mutually consistent experiences. This interpretation also explains why the result should not be reduced to “more publicity”: prior intervention evidence shows that information without convenient and concrete means of acting may have limited effects (Varotto and Spagnolli, 2017; Bernstad, 2014), while clear guidance can complement facilities and norms (Lange et al., 2014; Rousta et al., 2015; Wang et al., 2018; Li et al., 2023; Knickmeyer, 2020). Nevertheless, EP1 assessed confidence in knowledge rather than sorting accuracy, EP2 assessed exposure rather than message comprehension, and EP3 assessed conditional willingness rather than observed behavior. EP4 also met the Holm-adjusted criterion in the full-sample analysis, whereas EP5 did not, so broader family and community effects cannot be treated as equally established. EP5 did not meet the Holm-adjusted criterion, although its point estimate was positive. This result should therefore be interpreted as inconclusive rather than as evidence that community sorting rules were perceived identically in the two samples. The item assessed a conditional response concerning whether respondents believed they would sort better if their residential community had sorting rules, rather than actual exposure to such rules or observed behavioral change. Its relatively broad conditional framing may also have reduced its ability to distinguish between respondents who already experienced clear rules and those responding hypothetically. Given the modest sample size and multiplicity correction across 18 items, a moderate underlying difference cannot be excluded, but the present study does not provide sufficiently strong evidence to claim one. The Hong Kong result therefore identifies a comparatively coherent perceived information-to-action pathway, not proof that publicity caused superior sorting performance.

Chuzhou records showed lower mean responses and greater response-category diversity across EP1-EP4, accompanied by broader ordinal spread in the descriptive checks. This distributional pattern adds an important descriptive layer to the mean difference: it is compatible with some records reflecting adequate knowledge and publicity exposure while others reflected uncertainty or limited contact. In light of prior work, this could arise from uneven campaign reach, inconsistent labels, differences between university and residential communication channels, or variation in whether respondents could translate general messages into a concrete sorting decision (Varotto and Spagnolli, 2017; Bernstad, 2014; Knickmeyer, 2020). The present archive cannot distinguish among these possibilities. EP4 also met the Holm-adjusted criterion in the full-sample analysis, whereas EP5 did not. The evidence therefore does not support treating family participation and community rules as uniformly differentiated informational or social cues across the two samples. A candidate hypothesis for future research is that standardized bin labels, accessible station maps, category-specific examples, and immediate feedback may reduce informational unevenness more effectively than undifferentiated publicity. This proposition requires evaluation using objective knowledge tests, sorting-accuracy or contamination measures, and observed disposal behavior. In summary, Chuzhou’s principal EP signal is heterogeneous perceived access to usable sorting information, not simply weaker environmental motivation.

### 4.4 Economic returns and storage constraints motivate candidate practical hypotheses

The facilities-and-services results show why practical opportunity should be decomposed into external access, the household’s internal sorting workflow, service experience, time costs, and economic motives. These components can pull in different directions. Prior studies have linked shorter perceived distance and improved collection information to recycling (Lange et al., 2014; Rousta et al., 2015), and research combining norms with convenience found property-close collection to be independently associated with household sorting contributions (Berglund et al., 2022). Experimental field evidence further indicates that convenience inside the home can matter even when information is available (Bernstad, 2014). In a comparison across 10 OECD countries, environmental benefit and civic duty were prominent motivations, greater provision of collection services was associated with more recycling, and the design of monetary incentives mattered because poorly aligned incentives could displace voluntary moral motivation (Halvorsen, 2012). This literature makes the present EF pattern theoretically coherent: a person can perceive external facilities as accessible while still facing storage burdens at home, and economic return can be salient without being the sole or necessarily strongest basis for participation.

For the Hong Kong study area, EF3 indicated more favorable perceptions of facility labeling and proximity, whereas EF4 indicated a greater household storage-space burden; both contrasts met the Holm-adjusted criterion in the full-sample analysis. Rather than cancelling each other out, these results locate friction at different stages of the household recycling sequence. A nearby and clearly labeled collection point can reduce the cost of final disposal, but recyclables must first be separated, accumulated, and stored inside the dwelling. Bernstad’s field study is relevant because improvements to in-home sorting equipment changed observed separation when written information alone did not (Bernstad, 2014), and Hong Kong research has highlighted collection arrangements and the distinctive constraints of recycling in multi-family and high-rise settings (Yau, 2012; Ng, 2019). In the present data, the EF3-EF4 combination therefore motivates a “last-meter” hypothesis: perceived access outside the home may coexist with a bottleneck before materials leave the household. The non-significant EF2 and EF5 results further limit the scope of the practical-context interpretation. Both items showed modest between-sample point estimates, but neither met the Holm-adjusted significance criterion. Because the confidence intervals reported for Cliff’s delta are unadjusted descriptive intervals, their exclusion of zero does not override the multiplicity-adjusted inferential tests. With 51 records per sample, the study may have had limited precision for detecting modest effects after correction across 18 items; however, this does not establish that true differences exist. EF2 may also vary substantially with individual routines and perceived effort, while EF5 combines two related but distinct judgments, namely station proximity and service satisfaction, which may not vary in parallel. The results should therefore be treated as inconclusive rather than as evidence of either clear between-sample differences or definite equivalence. Housing type, floor area, objective distance, and pickup frequency were also unmeasured. Accordingly, the Hong Kong findings support testing compact in-home containers, reduced storage duration, or more frequent collection, while identifying coexisting external accessibility and internal spatial burden as the present study’s defensible conclusion.

The Chuzhou sample showed a substantially stronger economic-gain motive on EF1, while EF2 and EF5 did not meet the Holm-adjusted criterion. This pattern indicates that economic return was more psychologically salient, but it does not establish that participation was exclusively monetary, that records referred to the same kind of return, or that respondents actually sold materials or received formal rewards. The broader literature cautions against a simple rewards prescription. Cross-national evidence indicates that beliefs about environmental benefit and civic duty remain central and that incentive design should avoid crowding out voluntary motivation (Halvorsen, 2012); Hong Kong research also discusses economic incentives (Yau, 2010), showing that their relevance is not unique to mainland settings. In the present study, the stronger EF1 response is therefore best treated as one element of a mixed motivational profile. Future field experiments in Chuzhou could compare carefully specified deposit, points, or buy-back arrangements with information-only and convenience-enhancement conditions, while measuring whether any initial gain persists after rewards are withdrawn. Such designs should also test whether transparent rules and easy redemption complement, rather than replace, civic and environmental motives. The stronger EF1 response therefore identifies economic return as a comparatively salient self-reported motivation in the Chuzhou sample. It generates a candidate intervention hypothesis for subsequent behavioral testing rather than evidence that economic incentives would improve sorting.

### 4.5 Measurement boundaries and priorities for confirmatory comparative research

The measurement diagnostics strengthen the substantive interpretation by showing why the four labels should not be treated as interchangeable validated scales. Fifteen items came from Meng et al. (2019), three conditional items came from Xiao et al. (2017), and the resulting project item sets were not revalidated as a common four-factor structure. EA and SN showed limited and sample-dependent internal consistency, while the EF items combined economic benefit, time cost, external facilities, household storage, and service perceptions and produced a negative pooled alpha. These results do not support treating EA, SN, or EF as internally coherent composite scales in the present samples. EP alone showed high internal consistency, but its mixture of knowledge, publicity, and conditional cues means that alpha cannot establish unidimensionality (Sijtsma, 2009) or cross-context measurement invariance (Putnick and Bornstein, 2016). Accordingly, none of the four project item sets was analyzed as a composite construct. Item-level analysis was consequently not a fallback: it preserved theoretically meaningful contrasts that grouping-level scores could obscure or misrepresent.

The principal limitation of this study is its sampling scope. Each setting contributed only 51 complete questionnaire records from a single pragmatically selected university-community study area, and participants were recruited through a site-based, non-probability intercept procedure rather than through random or stratified population sampling. The two samples therefore cannot be considered representative of the broader populations of Hong Kong, Chuzhou, or mainland China. Moreover, because each geographical setting was represented by only one university-community study area, geographical location is inseparable from the specific local context, sample composition, and recruitment pathway. The observed between-sample differences should consequently be interpreted as site-specific exploratory patterns rather than as population-level regional differences. The present findings cannot establish whether the same response profiles would occur in other communities within Hong Kong or mainland China. Although comparison with larger urban studies can indicate whether particular psychological or contextual patterns are consistent with findings reported elsewhere, such convergence cannot establish the representativeness of the present non-probability samples.

The modest sample size also limits the precision of the item-level comparisons. With 51 records per study-area sample and Holm correction across 18 simultaneous tests, the present design is better positioned to identify comparatively large contrasts than to resolve small or moderate differences with precision. Confidence intervals for several smaller or moderate effect estimates remain wide enough to include both relatively small and potentially meaningful differences. Accordingly, failure to meet the Holm-adjusted significance criterion should be interpreted as insufficient evidence for a clear between-sample difference under the present design, not as evidence of similarity, absence of an effect, or statistical equivalence. The present study therefore emphasizes effect-size magnitude, confidence intervals, and response distributions alongside multiplicity-adjusted tests, while reserving confirmatory assessment of small and moderate contrasts for larger samples.

An additional limitation is the absence of individual-level demographic and socioeconomic covariates. Age, gender, education, income, occupation, housing type, and respondent role were not available in the archived analytic dataset. Therefore, the two study-area samples cannot be demonstrated to be comparable in demographic composition, and the observed between-sample differences may partly reflect unmeasured compositional differences rather than geographical context alone. In particular, possible differences in the proportions of students, university staff, and community residents cannot be evaluated retrospectively. The present analysis consequently does not estimate an independent regional effect after adjustment for participant characteristics.

A further analytic limitation concerns the recurrence of complete response patterns. The lower proportion of unique complete response patterns in the Chuzhou sample is descriptive and does not identify its psychological origin. Recurring response vectors could reflect genuinely similar perceptions among respondents, shared response tendencies, or concentration on common Likert-category combinations; they could also arise from other response processes that cannot be distinguished with the present archive. Because identical complete vectors are compatible with legitimate independent respondents, recurrence should not be interpreted as evidence of duplicate participation or data invalidity. The exploratory unique-pattern check is therefore not a validation procedure and does not provide independent evidence for or against the primary full-sample findings.

Future confirmatory research should recruit substantially larger samples from multiple universities, residential communities, and other population settings in each region. Future confirmatory studies should also prespecify sample-size or precision targets based on the smallest between-sample effects considered substantively important, so that small and moderate contrasts can be estimated with substantially narrower uncertainty intervals. Where feasible, probability-based or stratified sampling should be used to improve population coverage. Future studies should also collect detailed demographic, socioeconomic, occupational, and housing information and incorporate these variables into appropriately specified multivariable models to determine whether study-area differences remain after adjustment for sample composition. Larger, multi-site sampling will further be needed to distinguish individual-level compositional variation from contextual differences between locations. Such studies will be necessary to determine whether the item-level patterns identified in the present exploratory comparison are reproducible and generalizable beyond these two study areas.

## 5 Conclusion

Across the two study-area samples, strong endorsement of waste sorting’s resource-conservation value coexisted with different psychological and perceived-context profiles. Among the Holm-significant contrasts in the full-sample analysis, Hong Kong records reported greater sorting-and-station knowledge, perceived regulatory constraint, resident responsibility, publicity exposure, willingness under clear-label conditions, and perceived facility labeling and proximity. Chuzhou records, by contrast, showed stronger economic-gain motivation, while Hong Kong records reported a greater household storage-space burden. These findings distinguish a high resource-conservation endorsement in both samples from the normative, informational, motivational, and practical conditions through which that value may be translated into action.

These conclusions are restricted to the two university-community study-area samples examined in this study and should not be generalized to the broader populations of Hong Kong or mainland China. Larger, multi-site studies using more representative sampling strategies, ideally including probability-based or stratified recruitment and relevant demographic and contextual variables, are required to determine whether the present item-level patterns can be replicated and generalized at the population level.

Placed alongside the broader Hong Kong and mainland Chinese literature, the present item-level patterns generate candidate intervention hypotheses rather than establishing intervention effects. For the Hong Kong study area, subsequent behavioral research could test whether reducing household storage demands while maintaining visible normative and informational support changes observed sorting. For the Chuzhou study area, subsequent experiments could evaluate clearer labels, more accessible station information, and carefully specified economic-incentive conditions. These propositions remain hypotheses because the present study measured self-reported perceptions, motivations, and conditional willingness rather than observed sorting behavior or experimentally manipulated conditions. The item-level strategy was essential because weak or negative internal consistency within several original project item sets meant that composite scoring could have obscured these contrasting item-level patterns. More broadly, context-sensitive environmental-psychology assessment can translate the observed item-level patterns into specific, locally grounded hypotheses for subsequent behavioral testing.

## Data Availability

The raw data supporting the conclusions of this study are available from the corresponding author upon reasonable request.
