# Supplementary material for: High endorsement of resource conservation in both samples alongside divergent perceived waste-sorting contexts: an exploratory comparison of two university-community study areas in Hong Kong and mainland China
Source: Front Psychol. 2026 Oct 9;17:1968239. doi: 10.3389/fpsyg.2026.1968239 (PMC13690637; doi:10.3389/fpsyg.2026.1968239)
Supplement: Supplementary file 1 [file Supplementary_file_1.docx]

# Table S1. Reverse-worded items were reverse-scored as 6 minus the raw response for analyses requiring directionally aligned scoring.

| **Code** | **Project item set** | **English reporting translation of administered item** | **Reverse** | **Operational interpretation** | **Primary source** |
| --- | --- | --- | --- | --- | --- |
| **EA1** | EA | I am more concerned about livelihood and everyday-life issues than environmental issues such as waste recycling. | Yes | Relative priority of livelihood/everyday-life concerns over recycling-related environmental concerns | Meng et al. (2019) |
| **EA2** | EA | To conserve resources and protect the environment, I am willing to participate in waste sorting and recycling. | No | Self-reported willingness to participate in waste sorting and recycling | Meng et al. (2019) |
| **EA3** | EA | Waste sorting and recycling help conserve resources and turn waste into useful resources. | No | Endorsement of the resource-conservation benefit of waste sorting | Meng et al. (2019) |
| **EA4** | EA | I produce little recyclable waste and do not need to sort and recycle it. | Yes | Perceived lack of personal need to sort because little recyclable waste is produced | Meng et al. (2019) |
| **SN1** | SN | Participating in waste sorting and recycling gives me a sense of satisfaction. | No | Satisfaction associated with participating in waste sorting | Meng et al. (2019) |
| **SN2** | SN | Waste-sorting and recycling laws and regulations can constrain me. | No | Perceived constraint from waste-sorting laws and regulations | Meng et al. (2019) |
| **SN3** | SN | Waste sorting and recycling are the responsibility of government and businesses and have nothing to do with residents. | Yes | Attribution of sorting responsibility away from residents toward government and businesses | Meng et al. (2019) |
| **SN4** | SN | Seeing neighbours and friends participate in sorting would encourage me to do the same. | No | Conditional influence of neighbours’ or friends’ participation | Meng et al. (2019) |
| **EP1** | EP | I know how to sort waste and where nearby recycling stations are located. | No | Self-perceived knowledge of sorting methods and recycling-station locations | Meng et al. (2019) |
| **EP2** | EP | I have encountered sufficient publicity and education about waste sorting and recycling in daily life. | No | Perceived exposure to waste-sorting publicity and education | Meng et al. (2019) |
| **EP3** | EP | I would sort waste if the sorting labels on bins were clear. | No | Conditional willingness to sort when bin labels are clear | Xiao et al. (2017) |
| **EP4** | EP | I would sort waste better if my family members also participated. | No | Perceived facilitative influence of family-member participation | Xiao et al. (2017) |
| **EP5** | EP | I would sort waste better if my residential community had sorting rules. | No | Perceived facilitative influence of community sorting rules | Xiao et al. (2017) |
| **EF1** | EF | I sell scrap to obtain economic benefits. | No | Salience of economic return from selling recyclable materials | Meng et al. (2019) |
| **EF2** | EF | Waste sorting and recycling take too much time. | Yes | Perceived time burden of sorting and recycling | Meng et al. (2019) |
| **EF3** | EF | There are sorting bins and collection facilities in the community, with clear labels and short distances. | No | Perceived availability, labeling clarity, and proximity of community sorting facilities | Meng et al. (2019) |
| **EF4** | EF | Sorting and storing recyclables occupy substantial space in my home. | Yes | Perceived household storage-space burden | Meng et al. (2019) |
| **EF5** | EF | Recycling stations are close to home and recycling services are satisfactory. | No | Perceived recycling-station proximity and satisfaction with recycling services | Meng et al. (2019) |

Note. The questionnaire was administered in Chinese. The English text is a reporting translation; source labels identify item provenance, and the four project item-set labels were retained solely for coding and traceability rather than as validated psychometric dimensions, scales, or latent constructs.

# Table S2. Full item-level effects.

| **Item** | **Short label** | **HK M** | **CZ M** | **Cliff’s δ** | **95% CI** | **pHolm** |
| --- | --- | --- | --- | --- | --- | --- |
| **EA1** | Livelihood over environment | 3.29 | 2.31 | 0.411 | [0.205, 0.599] | .003 |
| **EA2** | Willingness to participate | 4.61 | 3.69 | 0.350 | [0.156, 0.536] | .007 |
| **EA3** | Resource-conservation value | 4.71 | 4.41 | 0.146 | [-0.042, 0.329] | .258 |
| **EA4** | Little waste; no need to sort | 3.12 | 3.20 | -0.045 | [-0.266, 0.176] | .694 |
| **SN1** | Participation satisfaction | 4.71 | 3.84 | 0.436 | [0.248, 0.609] | .00030 |
| **SN2** | Perceived regulatory constraint | 4.78 | 3.71 | 0.569 | [0.403, 0.719] | 7.05×10^-7^ |
| **SN3** | Residents not responsible | 4.20 | 3.37 | 0.321 | [0.121, 0.512] | .020 |
| **SN4** | Neighbour/friend participation cue | 4.59 | 3.88 | 0.312 | [0.114, 0.505] | .020 |
| **EP1** | Sorting and station knowledge | 4.75 | 3.16 | 0.596 | [0.428, 0.750] | 3.11×10^-7^ |
| **EP2** | Publicity/education exposure | 4.75 | 3.67 | 0.546 | [0.378, 0.705] | 3.08×10^-6^ |
| **EP3** | Sort if labels are clear | 4.76 | 3.51 | 0.518 | [0.340, 0.675] | 8.88×10^-6^ |
| **EP4** | Do better if family participates | 4.65 | 3.86 | 0.363 | [0.170, 0.545] | .005 |
| **EP5** | Do better if community has rules | 4.69 | 3.94 | 0.249 | [0.054, 0.434] | .065 |
| **EF1** | Economic-gain motivation | 2.43 | 3.67 | -0.537 | [-0.713, -0.341] | 2.30×10^-5^ |
| **EF2** | Time burden | 3.53 | 2.84 | 0.260 | [0.042, 0.476] | .084 |
| **EF3** | Perceived facility labels/proximity | 4.82 | 3.82 | 0.501 | [0.333, 0.659] | 8.97×10^-6^ |
| **EF4** | Household storage-space burden | 2.22 | 3.14 | -0.388 | [-0.577, -0.181] | .005 |
| **EF5** | Perceived station proximity/service | 3.92 | 3.27 | 0.236 | [0.015, 0.446] | .098 |

Note. HK = Hong Kong; CZ = Chuzhou; M = mean; pHolm = Holm-adjusted p value. Means and Cliff’s delta use directionally aligned scoring; positive delta indicates a higher aligned response in Hong Kong. Confidence intervals are unadjusted 10,000-resample percentile bootstrap intervals. Inferential status is based on Holm-adjusted two-sided Mann-Whitney tests across the 18 items.

# Table S3. Internal-consistency diagnostics for the original project item sets by sample.

| **Project item set** | **Sample** | **Raw Cronbach’s α** | **Bootstrap 95% CI** |
| --- | --- | --- | --- |
| **EA** | Overall | .483 | [.279, .628] |
| **EA** | Hong Kong | .603 | [.430, .723] |
| **EA** | Chuzhou | .322 | [−.154, .594] |
| **SN** | Overall | .642 | [.449, .774] |
| **SN** | Hong Kong | .653 | [.415, .800] |
| **SN** | Chuzhou | .404 | [−.186, .676] |
| **EP** | Overall | .919 | [.864, .952] |
| **EP** | Hong Kong | .860 | [.759, .924] |
| **EP** | Chuzhou | .895 | [.817, .941] |
| **EF** | Overall | −.168 | [−.566, .093] |
| **EF** | Hong Kong | −.204 | [−1.141, .244] |
| **EF** | Chuzhou | .092 | [−.291, .358] |

Note. Reverse-worded items were scored as 6 minus the raw response. All 10,000 record-level bootstrap resamples were valid. Alpha does not establish unidimensionality or measurement invariance.

# Figure S1. Cliff’s delta estimates for the full-sample analysis and the exploratory unique-pattern sensitivity analysis. Dark and light-blue points show the respective estimates; horizontal lines show 95% confidence intervals, and the vertical dashed line marks zero. Filled points indicate Holm-adjusted *p* < 0.05. In the unique-pattern analysis, one record per distinct complete response pattern was retained within each sample. Ten contrasts met the Holm-adjusted criterion: EA1, SN1–SN3, EP1–EP3, EF1, EF3, and EF4. These exploratory results do not validate or invalidate the primary findings.

**Figure S2**. Exploratory response-category diversity in the Hong Kong and Chuzhou samples. (A) Standardized Gini–Simpson index (*H*) by sample. (B) Difference in *H* (Hong Kong minus Chuzhou); the dashed line marks zero. Higher *H* indicates that responses occupy a larger or more even subset of the five response categories. Points show estimates, and horizontal lines show 10,000-resample bootstrap 95% confidence intervals. Filled points indicate Holm-adjusted significance in the exploratory permutation comparisons. Because *H* does not account for ordinal distance, it is not a measure of psychological polarization or ordinal response dispersion.
